# Traditional Preparations and Methanol Extracts of Medicinal Plants from Papua New Guinea Exhibit Similar Cytochrome P450 Inhibition

**DOI:** 10.1155/2016/7869710

**Published:** 2016-08-24

**Authors:** Erica C. Larson, Christopher D. Pond, Prem P. Rai, Teatulohi K. Matainaho, Pius Piskaut, Michael R. Franklin, Louis R. Barrows

**Affiliations:** ^1^Department of Pharmacology and Toxicology, University of Utah, 30 S. 2000 E., Salt Lake City, UT 84112, USA; ^2^School of Medicine and Health Sciences, University of Papua New Guinea, P.O. Box 5623, Boroko, NCD, Papua New Guinea; ^3^School of Natural and Physical Sciences, University of Papua New Guinea, P.O. Box 5623, Boroko, NCD, Papua New Guinea

## Abstract

The hypothesis underlying this current work is that fresh juice expressed from Papua New Guinea (PNG) medicinal plants (succus) will inhibit human Cytochrome P450s (CYPs). The CYP inhibitory activity identified in fresh material was compared with inhibition in methanol extracts of dried material. Succus is the most common method of traditional medicine (TM) preparation for consumption in PNG. There is increasing concern that TMs might antagonize or complicate drug therapy. We have previously shown that methanol extracts of commonly consumed PNG medicinal plants are able to induce and/or inhibit human CYPs* in vitro*. In this current work plant succus was prepared from fresh plant leaves. Inhibition of three major CYPs was determined using human liver microsomes and enzyme-selective model substrates. Of 15 species tested, succus from 6/15 was found to inhibit CYP1A2, 7/15 inhibited CYP3A4, and 4/15 inhibited CYP2D6. Chi-squared tests determined differences in inhibitory activity between succus and methanol preparations. Over 80% agreement was found. Thus, fresh juice from PNG medicinal plants does exhibit the potential to complicate drug therapy in at risk populations. Further, the general reproducibility of these findings suggests that methanol extraction of dried material is a reasonable surrogate preparation method for fresh plant samples.

## 1. Introduction

In Papua New Guinea (PNG) approximately 0.6% of the 7 million population is living with human immunodeficiency virus (HIV) [1] while over 30% [2] is infected with tuberculosis (TB). HIV is often a comorbidity of tuberculosis [2]. Standard treatment of HIV and TB requires long-term administration of antiretroviral (ARV) or anti-TB drugs. In PNG, traditional medicine (TM, bus marasin) is widely practiced [3], as is the use of Western medicines. In fact, in PNG it is thought that 50% of the population relies solely on traditional medicines, with no access to Western medicines [4]. Factors that influence use of traditional medicine for health promotion include cost of Western medicines, limited access to distribution centers, and poorly stocked aid posts and healthcare facilities in rural areas [5].

ARV and anti-TB therapies are often complicated by drug-drug interactions [6], most commonly via effects on drug metabolism performed by Cytochrome P450 enzymes (CYPs) present in the liver. CYPs are a superfamily of enzymes that metabolize drugs to a more-readily excreted form. Drugs interact with CYPs through two major mechanisms: enzyme inhibition and enhanced enzyme expression, termed induction [6]. In an effort to determine potential impact of traditional medicines on HIV/TB treatment efficacy, we previously assessed methanol extracts from 69 of the 100 most commonly used medicinal plants (as listed in UPNG Traditional Medicines Database [7, 8]), collected from 7 PNG provinces, for human CYP induction and inhibition* in vitro *[9]. Close to two-thirds of the medicinal plant extracts exhibited potent CYP inhibition, while almost one-third of them significantly induced CYP expression. This work confirmed that many PNG medicinal plants have the potential to induce or inhibit CYPs and may possibly interfere with the therapeutic efficacy of ARV and anti-TB drugs.

In PNG the most common method of medical plant preparation for consumption is to express the juice, often using a little water to facilitate capture [10]. Recent literature has compared fresh plant juice to alcohol extracts for antibiotic or antioxidant activity often demonstrating quantitative variation in the components isolated [11–18]. Such a comparison has not been made concerning drug metabolism, however, and the relevance of CYP inhibition by methanol extracts of medical plants to actual bus marasin practice has been questioned. It is important to resolve this question in PNG in order for the PNG National Aids Council to institute counselling of patients that receive ARV or TB therapy regarding the use of TMs. Data is presented here that shows that succus from several PNG medicinal plants can indeed inhibit human CYPs with 6 of 15 species inhibiting CYP1A2, 7/15 inhibiting CYP3A4, and 4/15 inhibiting CYP2D6. In addition, the data reveal that CYP inhibition observed in methanol extracts of dried plant material was by and large recapitulated in the succus preparations, with no statistically significant difference (*p* < 0.05) observed in their abilities to inhibit CYPs* in vitro*. Elements of this work were presented at the American Society of Pharmacognosy 2015 annual meeting [19].

## 2. Materials and Methods

### 2.1. Medicinal Plant Collection

A team from the University of Papua New Guinea (UPNG) consisting of trained botanists, pharmacognosists, and students interviewed traditional healers in the rural communities of Drekikir in the East Sepik Province, Dogura in the Milne Bay Province, Arawa and Namatoa in Bougainville, and National Capitol District. Fresh medicinal plant materials indicated in the traditional healer interview were collected, and expressed juices were prepared and refrigerated until return to the BioDiscovery laboratory at UPNG School of Medicine and Health Sciences, Port Moresby, for rotary evaporation. In accordance with our established protocols which require informed consent prior to interviews [8], plant information, including local names and medicinal uses, was documented. Voucher samples were prepared for plant identification by UPNG Biological Sciences Herbarium staff where vouchers are stored.

### 2.2. Medicinal Plant Extraction

Plant succus prepared from ~200 g fresh plant leaves was evaporated to dryness and dissolved in 100 mL of 100% methanol (MeOH) overnight. Extracts (50 mL) were then transferred to clean vessels, evaporated to dryness, and redissolved in dimethyl sulfoxide (DMSO) to yield a final concentration of approximately 10 mg/mL.

### 2.3. CYP Inhibition Assays

Inhibition assays were performed as previously described [9]. In brief, plant extracts were added to human liver microsomes enriched in their respective CYP subtype (Celsis). Substrate was added at half-saturating (*K*_*m*_) or saturating (*V*_max⁡_) concentrations. Substrates selective for their respective CYPs are as follows: methoxyresorufin (MR), CYP1A2; 7-benzyloxyquinoline (7-BQ), CYP3A4; and 7-methoxy-4-(aminomethyl)-coumarin (MAMC), CYP2D6. NADPH was added in excess (60 mM) and the formation of fluorescent product was measured using a Biotek-Synergy 2 Microplate Reader [20]. Extracts showing >50% inhibition at *K*_*m*_ and/or *V*_max⁡_ concentrations were deemed inhibitory (moderate to strong inhibitors as defined by FDA, 2012 [21]).

### 2.4. Statistics

To investigate statistical significance between TM preparation methods, data from the previous methanol study and current succus study was organized into a 2 × 2 contingency table and Chi-squared tests performed for each CYP subtype. Results were considered statistically significant when *p* < 0.05. Pairwise comparisons between of medicinal plants of like genus and species from the methanol study and current succus study were used to calculate percent agreement.

## 3. Results

### 3.1. CYP Inhibition by Medicinal Plant Succus

Succus was prepared in the field from 17 species of medicinal plants that were previously analysed for inhibition of CYPs [9]. Some of the plants were collected in more than one location. Overall, ~80% of CYP inhibitory activity found in the succus study was in agreement with the methanol study. CYP inhibition across the two studies organized by plant genus and species is summarized in Table 1. Juice from* Calophyllum inophyllum* L.,* Cassia alata* L.,* Casuarina equisetifolia* L.,* Passiflora foetida* L.,* Morinda citrifolia* L. (one of 2 collections), and* Terminalia catappa* L. was found to inhibit CYP 1A2. Plant succi that inhibited CYP 3A4 were* Calophyllum inophyllum* L.,* Cassia alata* L.,* Casuarina equisetifolia* L.,* Ipomea pes-caprae *L.,* Morinda citrifolia* L.,* Passiflora foetida* L.,* Sida rhombifolia* L., and* Terminalia catappa* L. For CYP 1A2 inhibition, only* Calophyllum inophyllum* L.,* Cassia alata* L.,* Passiflora foetida* L., and* Terminalia catappa* L. were active.

### 3.2. CYP Inhibition: Plant Succus Compared to Methanol Extracts

**Figure 1:**
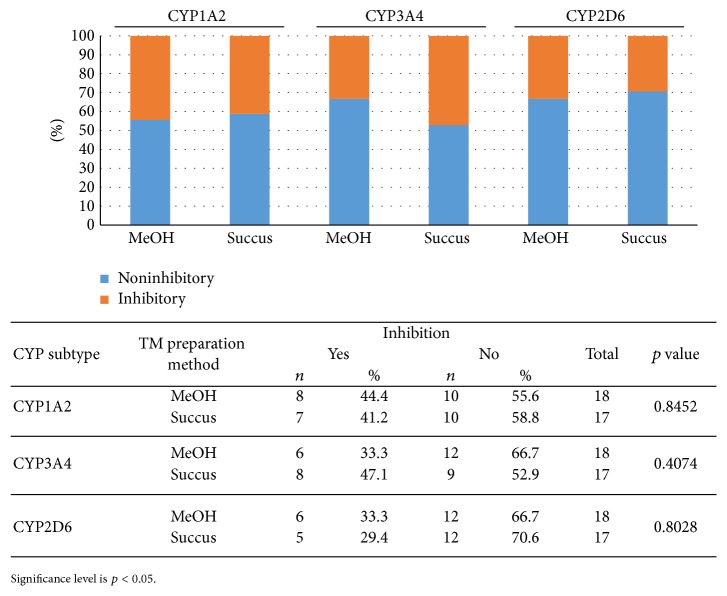
Comparison of CYP inhibitory activity based on TM preparation method.

Overall, CYP inhibitory activity (or lack thereof) determined for the succus plant preparations agreed with the previously determined inhibitory activity determined for the methanol preparations. For CYP1A2, out of 20 pairwise comparisons, nonagreement was found for 5:* Ipomea pes-caprae *L.,* Morinda citrifolia* L. (one of two preparations),* Passiflora foetida* L.,* Premna obtusifolia* R.Br.,* Sida rhombifolia* L., and* Terminalia catappa* L. For CYP3A4, out of 20 pairwise comparisons, nonagreement was found for 3:* Cassia alata* L. (two of two preparations) and* Sida rhombifolia* L. For CYP2D6, out of 20 pairwise comparisons, nonagreement was found for 4:* Bidens pilosa* L. (one of two preparations),* Casuarina equisetifolia* L., and* Passiflora foetida* L. (two of two preparations). Furthermore, several of the nonagreements were likely due to the magnitude of inhibition being close to the adopted “significant” cutoff of 50% for moderately active inhibitors [21]. Examples would be 2D6 inhibition of* Bidens pilosa *L. (51% inhibition at 40 *μ*M substrate), one of the two* Morinda citrifolia* L. samples (56% inhibition at 40 *μ*M substrate), and so forth. In any case, there was no statistical difference for CYP inhibition found in the succus study compared to the methanol study for CYP1A2, 3A4, or 2D6 (Figure 1; *p* < 0.05), overall agreement being approximately 84%. Therefore, methanol preparations may serve as a surrogate for traditional preparation methods, when fresh plant material is difficult to obtain.

## 4. Discussion

In PNG the most common method of medicinal plant preparation for oral consumption is squeezing of juice from the fresh plant (succus). In contrast, our previous work measured CYP inhibition by methanol extracts of dried medicinal plant parts. Therefore, this work investigated CYP inhibition by succus, the more traditional method preparation.

Antiretroviral and anti-TB therapies employ combinations of drugs to reduce disease burden. Metabolism of the majority of these drugs is performed by CYP3A4. Protease inhibitors (PIs) are the most common ARV component associated with drug-drug interactions. PIs inhibit CYP metabolism, a phenomenon which is often utilized to enhance pharmacokinetic profiles of other PIs taken concomitantly [22–24]. Common anti-TB drugs, such as isoniazid and rifapentine, have also been shown to cause CYP inhibition* in vitro* [25]. Excessive CYP inhibition can have negative consequences due to elevated concentrations of ARV or anti-TB drugs in the body [26, 27].

Traditional medicines have the potential to exacerbate adverse drug effects through plant-drug interactions. For instance, cat's claw,* Uncaria tomentosa* (Willd.), an herbal medicine taken to boost the immune system, has been observed to elevate protease inhibitor levels in the blood through CYP3A4 inhibition [28, 29]. Identification of herb-drug interactions, especially in countries where herbal medicines are widely practiced, is vital in order to avoid development of toxicity in patients receiving Western medicines for HIV and/or TB. Traditional medicine is widely practiced in Papua New Guinea (PNG). It is a likely scenario that traditional medicines will be used in combination with ARV and anti-TB drugs throughout much of PNG. Previously, we showed that around 40% of the PNG medicinal plant methanol extracts tested inhibited CYP3A4 [9]. In addition, CYP1A2 and CYP2D6, which are involved to a lesser extent with respect to ARV and anti-TB drug metabolism, were shown to be inhibited by around 28% and 29% of the PNG medicinal plant extracts tested, respectively.

Many factors can contribute to the irreproducibility of plant extract activity when extracts from independently collected plants of the same species are compared. It is well accepted that the chemical composition of recollected plant extracts can vary with location of collection, season of collection, varieties or cultivars sampled, and even age of the plant parts collected [30–34]. The solvent used for plant extraction can also greatly influence the chemical composition of the extract [35]. Recent work comparing juice to alcohol extracts has tended to indicate superior antibiotic activity in fresh juice [11–16], whereas superior antioxidant activity tended to follow extraction of flavones and phenols in alcohol or other preparations [17, 18]. In our previous work, which compared some plants that were independently recollected, the total percent agreement in CYP activity was reasonably good (91% for CYP induction and 72% for CYP inhibition) (unpublished data) and gave confidence that the activities identified in the methanol extracts of randomly collected dried plant samples were largely reproducible.

The ability of fresh juice from PNG medicinal plants to inhibit any of three human CYP enzymes was tested here in 17 samples from 15 species. Significant inhibition was determined in 7 of the 1A2 analyses, 8 of the 3A4 analyses, and 5 of the 2D6 analyses. Within this relatively limited dataset, statistical analysis could find no significant difference in the CYP inhibitory activity between the two extraction methods. This implies that the compounds likely responsible for drug-drug interaction appear to be, on average, equally accessed by traditional preparation of fresh plant material and by methanol extraction of dried material. The data indicate that methanol extraction of dried plant material is probably a reasonable surrogate preparation method when fresh plant samples cannot be obtained for analysis.

## Figures and Tables

**Table 1:** CYP inhibition across methanol (M) and succus (S) studies.

Genus and species	M/S	CYP1A2 % inhibition		Inh? (Y/N)	CYP3A4 % inhibition		Inh? (Y/N)	CYP2D6 % inhibition		Inh? (Y/N)
		MR (2.0 *μ*M)	MR (0.4 *μ*M)		7-BQ (500 *μ*M)	7-BQ (100 *μ*M)		MAMC (100 *μ*M)	MAMC (40 *μ*M)	
*Bidens pilosa * (Asteraceae)	M	—	—	N^#^	—	—	N^#^	—	—	N^#^
*Bidens pilosa * (Asteraceae)	M	—	—	N^#^	—	—	N^#^	—	51	Y^†^
*Bidens pilosa * (Asteraceae)	S	—	—	N^##^	—	—	N^##^	—	—	N^##^

*Calophyllum inophyllum * (Calophyllaceae)	M	74	86	Y^†^	—	79	Y^†^	52	60	Y^†^
*Calophyllum inophyllum * (Calophyllaceae)	S	61	86	Y^‡^	—	79	Y^‡^	70	—	Y^‡^

*Cassia alata* (Fabaceae)	M	—	54	Y^†^	—	—	N^#^	56	61	Y^†^
*Cassia alata* (Fabaceae)	M	58	75	Y^†^	—	57	Y^†^	54	—	Y^†^
*Cassia alata* (Fabaceae)	S	—	58	Y^‡^	—	62	Y^‡^	50	83	Y^‡^

*Casuarina equisetifolia* (Casuarinaceae)	M	66	65	Y^†^	54	61	Y^†^	—	54	Y^†^
*Casuarina equisetifolia* (Casuarinaceae)	S	70	80	Y^‡^	—	74	Y^‡^	—	—	N^##^

*Eleusine indica* (Poaceae)	M	—	—	N^#^	—	—	N^#^	—	—	N^#^
*Eleusine indica* (Poaceae)	M	—	—	N^#^	—	—	N^#^	—	—	N^#^
*Eleusine indica* (Poaceae)	S	—	—	N^##^	—	—	N^##^	—	—	N^##^

*Ficus wassa* (Moraceae)	M	—	—	N^#^	—	—	N^#^	—	—	N^#^
*Ficus wassa* (Moraceae)	S	—	—	N^##^	—	—	N^##^	—	—	N^##^

*Hibiscus rosa-sinensis* (Malvaceae)	M	—	—	N^#^	—	—	N^#^	—	—	N^#^
*Hibiscus rosa-sinensis* (Malvaceae)	S	—	—	N^##^	—	—	N^##^	—	—	N^##^

*Hibiscus tiliaceus* (Malvaceae)	M	—	—	N^#^	—	—	N^#^	—	—	N^#^
*Hibiscus tiliaceus* (Malvaceae)	S	—	—	N^##^	—	—	N^##^	—	—	N^##^

*Ipomoea pes-caprae* (Convolvulaceae)	M	56	69	Y^†^	58	60	Y^†^	—	—	N^#^
*Ipomoea pes-caprae* (Convolvulaceae)	S	—	—	N^##^	—	70	Y^‡^	—	—	N^##^

*Laportea decumana* (Urticaceae)	M	—	—	N^#^	—	—	N^#^	—	—	N^#^
*Laportea decumana* (Urticaceae)	S	—	—	N^##^	—	—	N^##^	—	—	N^##^

*Morinda citrifolia* (Rubiaceae)	M	—	—	N^#^	—	—	N^#^	—	—	N^#^
*Morinda citrifolia* (Rubiaceae)	S	—	56	Y^‡^	—	—	N^##^	—	—	N^##^
*Morinda citrifolia* (Rubiaceae)	S	—	—	N^##^	—	—	N^##^	—	—	N^##^

*Passiflora foetida* (Passifloraceae)	M	—	—	N^#^	—	51	Y^†^	—	—	N^#^
*Passiflora foetida* (Passifloraceae)	S	—	52	Y^‡^	—	59	Y^‡^	—	52	Y^‡^
*Passiflora foetida* (Passifloraceae)	S	—	64	Y^‡^	—	69	Y^‡^	55	62	Y^‡^

*Premna obtusifolia* (Verbenaceae)	M	53	68	Y^†^	—	—	N^#^	—	—	N^#^
*Premna obtusifolia* (Verbenaceae)	S	—	—	N^##^	—	—	N^##^	—	—	N^##^

*Sida rhombifolia* (Malvaceae)	M	54	75	Y^†^	—	—	N^#^	—	—	N^#^
*Sida rhombifolia* (Malvaceae)	S	—	—	N^##^	—	60	Y^‡^	—	—	N^##^

*Terminalia catappa* (Combretaceae)	M	96	92	Y^†^	64	80	Y^†^	67	81	Y^†^
*Terminalia catappa* (Combretaceae)	S	84	76	Y^‡^	73	87	Y^‡^	55	68	Y^‡^

Inhibition indicated by “†” (for M study) and“‡” (for S study). No inhibition indicated by “#” (for M study) and“##” (for S study).

(—) activity was <50% inhibition.
